# Characterising protein/detergent complexes by triple-detection size-exclusion chromatography

**DOI:** 10.1186/s12575-015-0031-9

**Published:** 2016-02-15

**Authors:** Katharina Gimpl, Jessica Klement, Sandro Keller

**Affiliations:** Molecular Biophysics, University of Kaiserslautern, Erwin-Schrödinger-Str. 13, 67663 Kaiserslautern, Germany

**Keywords:** Membrane proteins, Detergent micelles, Absolute mass determination, Multiple detection, Static light scattering

## Abstract

**Background:**

In vitro investigations of membrane proteins usually depend on detergents for protein solubilisation and stabilisation. The amount of detergent bound to a membrane protein is relevant to successful experiment design and data analysis but is often unknown. Triple-detection size-exclusion chromatography enables simultaneous separation of protein/detergent complexes and protein-free detergent micelles and determination of their molar masses in a straightforward and absolute manner. Size-exclusion chromatography is used to separate different species, while ultraviolet absorbance, static light scattering, and refractive index measurements allow molar mass determination of protein and detergent components.

**Results:**

We refined standard experimental and data-analysis procedures for challenging membrane-protein samples that elude routine approaches. The general procedures including preparatory steps, measurements, and data analysis for the characterisation of both routine and complex samples in difficult solvents such as concentrated denaturant solutions are demonstrated. The applicability of the protocol but also its limitations and possible solutions are discussed, and an extensive troubleshooting section is provided.

**Conclusions:**

We established and validated a protocol for triple-detection size-exclusion chromatography that enables the inexperienced user to perform and analyse measurements of well-behaved protein/detergent complexes. More experienced users are provided with an example of a more sophisticated analysis procedure allowing mass determination under challenging separation conditions.

## Background

Membrane proteins are of outstanding biological and pharmacological relevance [1], but progress in their biophysical, biochemical, and structural investigation is hampered by the fact that these hydrophobic proteins are generally soluble in aqueous solution only in the presence of detergent micelles or other membrane-mimetic systems [2]. Upon solubilisation, various and sometimes large amounts of detergent are associated with the membrane protein of interest, and such protein/detergent complexes (PDCs) coexist with (mixed) micelles and detergent monomers [3]. The composition of PDCs depends drastically on the type and concentration of detergent used [3–5], so that the choice of detergent and its concentration has a great influence on the protein’s structure, stability, and functionality [6]. Membrane proteins tend to aggregate and precipitate if the detergent concentration is too low; conversely, excess detergent may lead to denaturation or dissociation of protein complexes [7, 8]. Therefore, detailed knowledge of detergent concentration and PDC composition is essential for many functional and structural studies [3, 9]. Likewise, biophysical investigations into membrane-protein folding based on the use of chemical denaturants require in-depth knowledge of the aggregational state of the protein/detergent or protein/lipid mixture under both native and denaturing conditions [10, 11]. In experiments relying on the chemical unfolding of a detergent-solubilised membrane protein, the unfolded polypeptide in the presence of high denaturant concentrations serves as a common reference state enabling the comparison of protein conformational stability among different detergents, but this approach is applicable only if the unfolded state is not associated with detergent [12].

### Experimental methods for determining molar masses

Experimental determination of the molar mass and oligomeric state is an essential step in the biophysical characterisation of proteins and protein complexes. Sodium dodecyl sulphate polyacrylamide gel electrophoresis (SDS-PAGE) is often used to identify proteins during the purification process and confirm their molar masses [13, 14]. However, membrane proteins usually reveal a migration behaviour different from that of soluble proteins, thus impeding mass determination by standard SDS-PAGE [15]. Size-exclusion chromatography (SEC) can yield valuable information on sample homogeneity and oligomeric state of water-soluble proteins but is of limited use for detergent-solubilised proteins. Mass estimation by SEC is based on a comparison of the elution behaviour of the protein of interest with that of standard proteins, which are globular and water-soluble. Since detergent binding substantially alters a protein’s elution behaviour [16] and, thus, its apparent size, analysing membrane proteins with SEC will provide, at best, a very crude size estimate. Nevertheless, with careful sample preparation, information on the coexistence of different oligomeric species can be deduced from SEC elution profiles even without knowledge of exact masses [5]. Hence, SEC is widely used for the qualitative analysis of PDCs, for example, to check homogeneity, stability, and purity of PDCs for use in structural studies such as crystallography [17, 18]. As a complementary technique, dynamic light scattering (DLS) provides information on the hydrodynamic radius of a PDC and identifies aggregates [19, 20]. Neither SEC nor DLS, however, can distinguish the contributions of detergent and protein components to the overall hydrodynamic behaviour of the complex. A well-established method to overcome this problem is analytical ultracentrifugation (AUC) [21–23], which combines separation of different species with thermodynamic analysis and, thus, is particularly suitable for multicomponent systems [24] such as detergent micelles and PDCs [25]. Drawbacks of AUC, however, comprise long experimental timescales of several hours up to a day, which restricts its applicability to relatively stable proteins [26], and difficulties encountered with floating detergents such as lauryldimethylamine *N*-oxide (LDAO), which elude analysis by this method [27]. Moreover, AUC is an expensive method, and sample preparation, measurements proper, and data analysis require an experienced user [27].

**Fig. 1:**
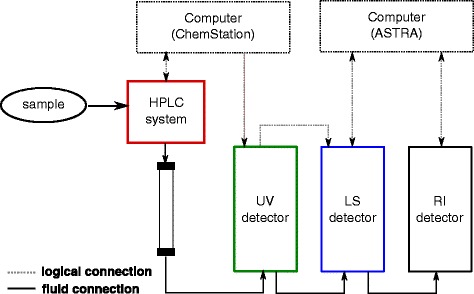
Schematic setup of triple-detection SEC. A high-performance liquid chromatography (HPLC) system is used to provide constant flow and an SEC column to separate different species. UV absorbance and RI detectors monitor changes in protein and detergent concentrations, while an LS detector follows changes in scattering intensity at multiple scattering angles. Data acquisition is controlled by a software package

By contrast, triple-detection SEC is a more straightforward, cheaper, and more widely available method that is exquisitely suited for determining the masses of both protein and detergent components and, consequently, PDC composition. In this method, SEC is coupled to a triple-detector system consisting of ultraviolet (UV) absorbance, static light scattering (LS), and refractive index (RI) detection, where separation and analysis are combined in a single experimental setup. Figure 1 shows a schematic setup with SEC being used exclusively for the preparative separation of different species such as detergent micelles and PDCs but not for any analytical purposes, in particular, determination of molar masses. Thus, in contrast with classical SEC approaches, quantitative analysis does not rely on elution volumes, which eliminates the need for calibration. Instead, the LS signal provides information on the molar masses of all scattering particles eluted in the course of an SEC run according to the equation [28]:

1 $$ \varDelta {R}_{\theta }={R}_{\theta, \mathrm{sample}}-{R}_{\theta, \mathrm{solvent}}=K{M}_wc $$

Here, *R*_*θ*_ is the Rayleigh ratio, which is defined as the total intensity of scattered light observed at a scattering angle *θ* at distance *r* from the point of scattering, normalised with respect to the scattering volume *V* and the intensity of incident light *I*_0_. Δ*R*_*θ*_ refers to the excess Rayleigh ratio, that is, the Rayleigh ratio of the sample (*R*_*θ*,sample_) minus that of the solvent (*R*_*θ*,solvent_). Furthermore, *K* is an optical constant, *M*_*w*_ the weight-average (or, more precisely, mass-average) molar mass of the scattering particle in solution or suspension, and *c* its concentration. Note that, in the so-called Rayleigh scattering regime (i.e., for small particles with a diameter below ~ *λ*_0_/20, where *λ*_0_ is the wavelength used), light scattering is isotropic, so that Δ*R*_*θ*_ is independent of the detection angle.

### Basic light scattering theory

It is important to keep in mind that Equation (1) is valid only for small particles at low concentrations, as it is a special form of the general Zimm relationship [29]:

2 $$ \Delta {R}_{\theta } = Kc\left({M}_w{P}_{\theta }+\frac{1}{2{A}_2c}+\dots \right) $$

Here, *P*_*θ*_ is the so-called form factor, which reflects the angular dependence of the scattering intensity, and *A*_2_ is the second virial coefficient, which is the first and most important term accounting for interparticle interactions. The optical constant *K* is given by

3 $$ K=\frac{4{\uppi}^2{n}_0^2{\left(\frac{\mathrm{d}n}{\mathrm{d}c}\right)}^2}{N_{\mathrm{A}}{\lambda}_0^4} $$

with *n*_0_ being the solvent’s refractive index, d*n*/d*c* the refractive index increment of the scattering particle in a given solvent, and *N*_A_ Avogadro’s constant. Coupling static light scattering with SEC leads to dilution of the sample to concentrations at which intermolecular interactions and, thus, the second term in parenthesis on the right-hand side of Equation (2) can safely be neglected. Moreover, scattering by proteins with molar masses <10^6^ g/mol is isotropic; therefore, the observed scattering intensity is angle-independent, and the form factor approaches unity, which further simplifies Equation (2) to the form given in Equation (1). For membrane proteins solubilised in detergent micelles, the experimentally accessible values of the refractive index increment, the concentration, and the molar mass refer to the entire PDC (comp). These quantities are related to those of the pure protein (prot) and detergent (det) through the following equations:

4 $$ {M}_{w,\mathrm{comp}}=\left(1+\delta \right){M}_{w,\mathrm{prot}} $$

5 $$ {c}_{\mathrm{comp}}=\left(1+\delta \right){c}_{\mathrm{prot}}=\frac{\Delta {\mathrm{UV}}_{280\mathrm{nm}}}{\left(\left(\frac{1}{1+\delta}\right){A}_{280\mathrm{nm},\mathrm{prot}}+\left(\frac{\delta }{1+\delta}\right){A}_{280\mathrm{nm}, \det}\right)} $$

and

6 $$ {\left(\frac{\mathrm{d}n}{\mathrm{d}c}\right)}_{\mathrm{comp}}=\frac{\Delta \mathrm{R}\mathrm{I}}{{\Delta \mathrm{U}\mathrm{V}}_{280\mathrm{nm}}}\left(\left(\frac{1}{1+\delta}\right){A}_{280\mathrm{nm},\mathrm{prot}}+\left(\frac{\delta }{1+\delta}\right){A}_{280\mathrm{nm}, \det}\right) $$

where *δ* is the mass ratio of bound detergent to protein (in g/g), ΔUV_280nm_ is the background-corrected UV absorbance signal, *A*_280nm_ is the extinction coefficient (in mL/(g cm)), and ΔRI is the background-corrected (i.e., the excess) refractive index signal. Generally, *δ* is not known but can be calculated from measured quantities on the basis of the refractive index values of the protein and the detergent, (d*n*/d*c*)_prot_ and (d*n*/d*c*)_det_, respectively, according to

7 $$ \delta =\frac{\left(\raisebox{1ex}{$\Delta \mathrm{R}\mathrm{I}{A}_{280\mathrm{nm},\mathrm{prot}}$}\!\left/ \!\raisebox{-1ex}{$\Delta \mathrm{U}\mathrm{V}$}\right.\right)-{\left(\frac{\mathrm{d}n}{\mathrm{d}c}\right)}_{\mathrm{prot}}}{{\left(\frac{\mathrm{d}n}{\mathrm{d}c}\right)}_{\det }-\left(\raisebox{1ex}{$\Delta \mathrm{R}\mathrm{I}{A}_{280\mathrm{nm}, \det }$}\!\left/ \!\raisebox{-1ex}{$\Delta \mathrm{U}\mathrm{V}$}\right.\right)} $$

With the aid of Equation (2), the molar mass of the PDC can be determined, and subsequent calculation of *δ* allows decomposition into protein and detergent contributions [28, 30].

### Triple-detection SEC

As outlined above, the combination of SEC as a separation technique with LS as an absolute technique for molar mass determination raises light scattering to a new qualitative level by allowing determination not only of the average molar mass of a sample but of the individual masses of all species that elute at different time points within a single measurement [30]. In particular, the combination of LS detection with the differential sensitivities of UV absorbance and RI detection towards detergent and protein concentrations enables determination of the contributions of each component (i.e., protein and detergent) to the overall LS signal to yield the composition of PDCs [28]. After system equilibration, which needs no attendance of the experimenter, a single measurement is performed within 1 h. Triple-detection SEC has become particularly popular in the field of membrane-protein analysis, where several publications on the application of triple-detection SEC to PDCs have been published over the past few years [11, 28, 31–34]. In the following, we describe a detailed procedure that is applicable even to complex samples such as membrane proteins in the presence of both detergent and high denaturant concentrations.

### Sample data

To demonstrate the general experimental setup as well as preparatory steps and data analysis, we present a basic characterisation of outer membrane phospholipase A (OmpLA) from *Escherichia coli* solubilised in the zwitterionic detergent LDAO. This β-barrel membrane protein was recombinantly produced in *E. coli* as inclusion bodies and subsequently refolded into LDAO micelles, but the protocol described here can be adapted to any detergent-solubilised membrane protein. For instance, we have successfully applied this protocol to the α-helical membrane protein Mistic from *Bacillus subtilis* solubilised in the nonionic detergents *n*-dodecyl-β-D-maltoside (DDM) and *n*-nonyl-β-D-maltoside (NM) to demonstrate that urea-induced unfolding in the presence of either of the two detergents results in an unfolded state that is dissociated from micelles irrespective of the detergent used [11]. More results obtained for Mistic solubilised in homologous alkyl maltoside detergents are shown and discussed below, where more advanced analysis procedures and limitations of the technique are also demonstrated.

## Results and discussion

### Separation and characterisation of PDCs and protein-free micelles

**Fig. 2:**
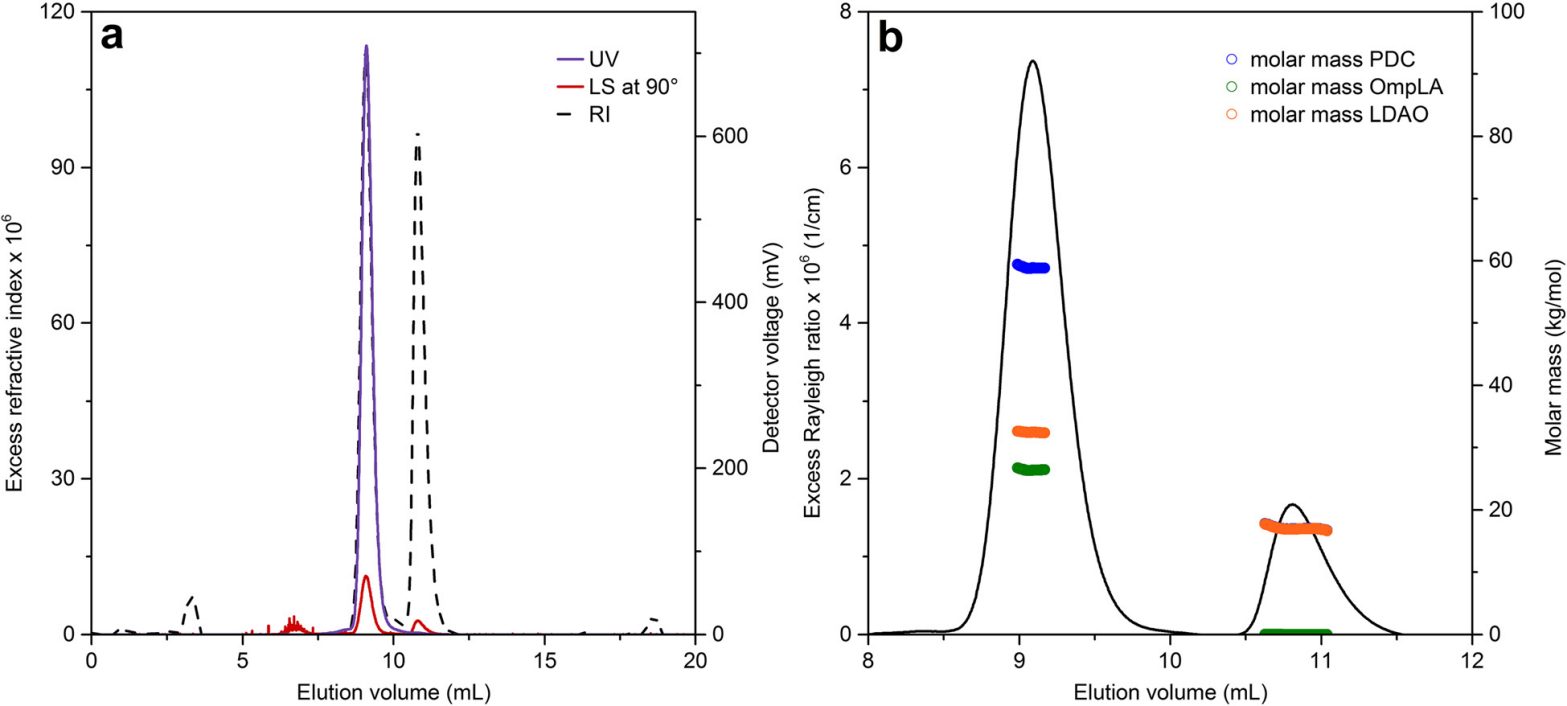
**a** Elution profile and **b** molar masses of OmpLA/LDAO complexes. **a** Excess RI values and voltages of UV and LS detectors are depicted as functions of elution volume. PDCs and protein-free detergent micelles elute as two separate peaks at 9 mL and 11 mL, respectively. Peaks at 3 mL and 7 mL are so-called “system peaks” caused by injected air or shedding of the SEC column. **b** Excess Rayleigh ratios at a scattering angle of 90° and derived molar masses of OmpLA, LDAO, and the OmpLA/LDAO PDC are plotted versus elution volume. 3 mg/mL OmpLA in 50 mM Tris, 100 mM KCl, 2 mM EDTA, pH 8.3, *c*_det,buffer_ = 4 mM, *c*_det,sample_ = 5 mM, flow rate 0.4 mL/min, room temperature (RT)

Figure 2a shows the elution profile of monomeric OmpLA refolded into LDAO [35] as monitored by UV absorbance, LS, and RI detectors. Data were recorded according to the procedure described in the protocol below. The elution profile reveals two distinct peaks at ~9 mL and ~11 mL. Strong signals in all three detectors identify the first peak as the one containing protein, whereas the absence of a noticeable 280-nm absorbance signal indicates that the second peak reflects protein-free LDAO micelles. This demonstrates that SEC can separate both species with baseline resolution, meaning that the first peak returns to baseline level before the second peak rises, thus allowing for straightforward and independent determination of the molar masses and compositions of both species (see Fig. 2b). The best-fit value of the weight-average molar mass of OmpLA is 28 kg/mol, which is in good agreement with the nominal mass of 31 kg/mol of the monomeric protein. In our experience, deviations from the expected value of ±10 % are typical of complex membrane-protein samples, although even better agreement may be obtained for very well-behaved systems [11]. The mass ratio of bound LDAO was calculated as 1.15 g/g, meaning that 1.15 g detergent is bound per 1 g protein. This yields a detergent contribution of 33 kg/mol to the overall molar mass of the PDC, which amounts to 61 kg/mol. Analysis of the protein-free peak yielded a molar mass of 17 kg/mol for empty LDAO micelles, thus reproducing the results for micellar LDAO measured in the absence of OmpLA (data not shown). These results represent a typical well-behaved membrane protein/detergent mixture comprising two species that can be neatly separated by SEC. In such cases, triple-detection SEC is an excellent method for determining both the oligomeric state of the protein and the amount of detergent bound to it.

### Systematic investigation of the influence of urea on micelle size

**Fig. 3:**
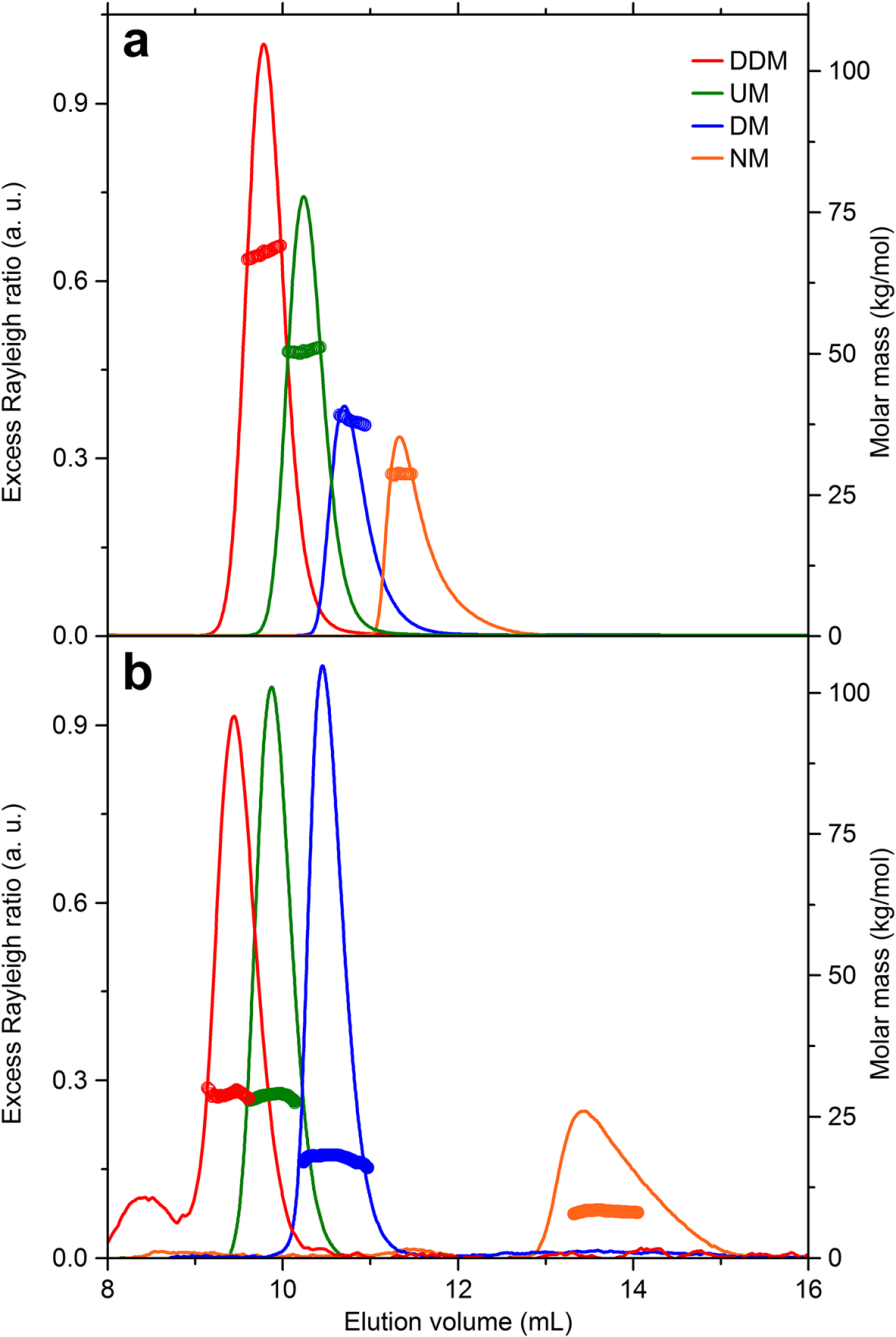
SEC as monitored by static light scattering of different alkyl maltosides in the (**a**) absence and (**b**) presence of 6 M urea. Excess Rayleigh ratios at 90° and molar masses of DDM, UM, DM, and NM are depicted as functions of elution volume. 50 mM Tris, pH 7.4, 50 mM NaCl, *c*_det,sample_ = CMC_8 M urea_ + 5 mM, *c*_det,buffer_ = CMC_buffer_ + 2 mM, flow rate 0.4 mL/min, RT

Co-solvents such as urea can drastically change the micellisation behaviour of detergents. For alkyl maltosides, for example, it has been shown by DLS that the micelle size decreases in the presence of denaturant [36]. However, size determination by DLS provides only the *z*-average hydrodynamic radius of scattering particles, which, additionally, is influenced by hydration of detergent headgroups. Since hydration is also affected by denaturants, a decrease in hydrodynamic particle size could result from changes in headgroup hydration and/or intrinsic micelle size. By contrast, mass information deduced from static light scattering is less sensitive to hydration effects; thus, changes in molar mass can unambiguously be ascribed to changes in micellisation behaviour. Figure 3 depicts normalised excess Rayleigh ratios and corresponding molar masses of alkyl maltosides carrying chains comprising 9–12 carbon atoms, namely, *n*-dodecyl-β-D-maltoside (DDM), *n*-undecyl-β-D-maltoside (UM), *n*-decyl-β-D-maltoside (DM), and *n*-nonyl-β-D-maltoside (NM). In these experiments, 100 μL running buffer containing 5 mM micellar detergent (i.e., detergent at a total concentration corresponding to the critical micellar concentration (CMC) plus 5 mM) was injected into the triple-detection SEC setup. The chromatography buffer contained 2 mM micellar detergent to avoid demicellisation resulting from dilution upon injection of the sample.

Figure 3a shows micelles in the absence and Fig. 3b micelles in the presence of 6 M urea. Without urea, molar mass determination yields 66 kg/mol for DDM, 50 kg/mol for UM, 38 kg/mol for DM, and 26 kg/mol for NM. The masses derived in the presence of 6 M urea are significantly smaller with 29 kg/mol, 28 kg/mol, 18 kg/mol, and 7 kg/mol, respectively. Despite the pronounced size reduction caused by urea, the elution volumes decreased under denaturing conditions for all detergents except NM. Hence, classical SEC analysis based solely on elution volumes would have been possible, in the optimal case, only after column calibration in the presence of 6 M urea. Under denaturing conditions, bending or sloping of the molar mass across the peak, as observed, for instance, for DM in Fig. 3b, is caused by instabilities in the RI baseline. At high denaturant concentrations, the RI baseline tends to oscillate, thus interfering with baseline correction. In such cases, the RI signal becomes distorted with respect to the LS signal, leading to inaccuracies in the determination of concentrations, which, in turn, results in slightly varying masses across the peak. Notwithstanding this issue, the average molar mass value across the entire peak is usually still reliable. With triple-detection SEC, scattering particles can be analysed without additional calibration even in complex situations such as the presence of high denaturant concentrations. Contrary to previous claims [37], these results demonstrate that triple-detection SEC is also compatible with the use of detergents and high concentrations of chemical denaturants in general.

### Characterisation of the membrane protein Mistic in detergent micelles under native conditions

**Fig. 4:**
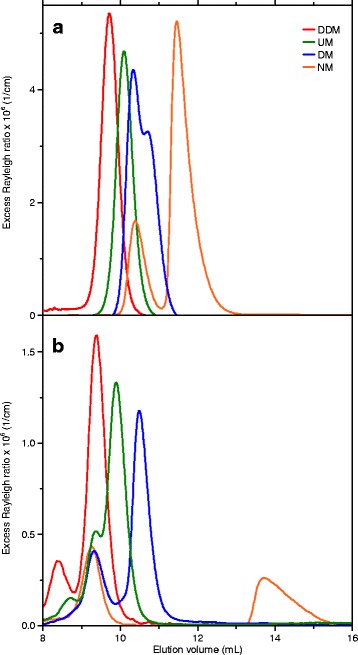
SEC as monitored by static light scattering of Mistic solubilised in different alkyl maltosides in the (**a**) absence and (**b**) presence of 6 M urea. **a** Excess Rayleigh ratios at 90° for Mistic solubilised in DDM, UM, DM, and NM are depicted as functions of elution volume. In the absence of urea, the traces of NM and DM show two peaks, indicating separation of PDCs and protein-free micelles. Single peaks for UM and DDM indicate co-elution of PDCs and micelles. **b** At 6 M urea, for all detergents except DDM, unfolded protein and detergent micelles are separated. The elution volume of the protein is at ~9.3 mL for all measurements, whereas the elution volumes of detergent micelles change according to their size. For DDM, unfolded detergent-free Mistic and DDM micelles co-elute [11]. Additional peaks in the SLS signal at ~8.5 mL in the DDM and UM traces are so-called “system peaks”; these peaks are typically caused by injected air or shedding of the SEC column and do not show up in UV and RI signals. 1 mg/mL Mistic, 50 mM Tris, pH 7.4, 50 mM NaCl, *c*_det,sample_ = CMC_8 M urea_ + 5 mM, *c*_det,buffer_ = CMC_buffer_ + 2 mM, flow rate 0.4 mL/min, RT

Another example of PDC analysis with the aid of triple-detection SEC is the α-helical membrane protein Mistic from *Bacillus subtilis* solubilised in different alkyl maltoside detergents [11]. In each experiment, 100 μL running buffer containing 78 μM Mistic and 5 mM micellar detergent was injected into the triple-detection SEC setup. As in the protein-free detergent measurements described above, the chromatography buffer contained 2 mM micellar detergent. Complete sample preparation, including recombinant production and chromatographic purification of the protein, is described in more detail elsewhere [11]. Figure 4a shows excess Rayleigh ratios of Mistic in alkyl maltosides with chains having 9–12 carbon atoms under native conditions, that is, in the absence of denaturant. As in the case of protein-free alkyl maltoside micelles (see above), the elution volume of the PDC depends on the alkyl chain length, with smaller complexes eluting later (see Table 1).

**Table 1:** Overview of elution volumes and molar masses of Mistic/alkyl maltoside complexes under native and denaturing conditions

0 M urea				
Sample	Elution volume (mL)	*M*_*w*,comp_ (kg/mol)	*M*_*w*,prot_ (kg/mol)	*M*_*w*,det_ (kg/mol)
Mistic + DDM	9.8	53	13	40
Mistic + UM	10.0	47	13	34
Mistic + DM	10.3	33	13	20
	10.8	38	0	38
Mistic + NM	10.5	25	13	12
	11.5	28	0	28
6 M urea				
Sample	Elution volume (mL)	*M*_*w*,comp_ (kg/mol)	*M*_*w*,prot_ (kg/mol)	*M*_*w*,det_ (kg/mol)
Mistic + DDM	9.3	0	13*	30*
Mistic + UM	9.3	12	12	0
	10.0	23	0	23
Mistic + DM	9.3	13	13	0
	10.8	19	0	19
Mistic + NM	9.3	13	13	0
	13.5	9	0	9

*Values derived from linear combination of independent Mistic and DDM measurements rather than global analysis

For Mistic/NM, analysis is straightforward and can be performed as described in this protocol, yielding molar masses of 13 kg/mol and 12 kg/mol for Mistic and bound NM, respectively. The experimental protein molar mass agrees very well with the nominal monomeric mass of 12.8 kg/mol. Analysis of the second, nicely separated peak yields a molar mass of 28 kg/mol, which is in good agreement with pure NM micelles (see above). Similarly, the molar mass of Mistic was identified as 13 kg/mol also for all of the other examples presented in Fig. 4a (see Table 1). However, the mass ratio of bound detergent/protein appears to increase from ~1 g/g for the Mistic/NM complex to ~2 g/g for Mistic/DM and further to ~3 g/g for Mistic/DDM, indicating overestimation of the amount of bound detergent for the long-chain detergents. This can be explained by poor separation by SEC of these PDCs from the corresponding protein-free micelles. If incomplete separation occurs, contributions from different species to the signals cannot be separated out neatly, and data analysis requires more caution. In the case of DM, PDCs and protein-free micelles are only partially separated but can still be analysed separately, provided that the peak boundaries are set such that the overlap between the two peaks is minimised. By contrast, elution profiles in the presence of UM or DDM give rise to only one peak. Even in the above cases, the molar mass and the oligomeric state of the protein can still be estimated reasonably. However, the amount of bound detergent becomes difficult or impossible to quantify because triple-detection SEC averages masses across all species present in the range analysed. In particular, the detergent concentration derived from the RI signal is the sum of bound detergent and detergent co-eluting in the form of protein-free micelles, resulting in overestimation of the amount of detergent bound to the protein.

### Limitations of standard analysis procedures and solutions

Triple-detection SEC of Mistic in the presence of 6 M urea allowed us to demonstrate that the protein’s unfolded state is detergent-free and may serve as a common reference state for protein-folding studies [11]. At high urea concentrations, baseline instabilities prevent reliable data analysis. Even at 6 M urea, baseline instabilities, particularly in the RI signal, can impede baseline subtraction in the ASTRA software. Therefore, elution profiles of Mistic under denaturing conditions were analysed using a customised analysis script allowing more sophisticated baseline-subtraction and data-analysis procedures. For most alkyl maltosides, quantitative analysis based on light scattering theory was straightforward, yielding a protein mass of 12–13 kg/mol for the peak at ~9.3 mL in Fig. 4b and demonstrating the absence of detergent from this peak. Hence, this peak represents unfolded Mistic without any bound detergent. Additionally, analysis of the peaks eluting at higher volumes yielded masses very close to those observed in measurements of the corresponding detergents without protein, namely, 23 kg/mol for UM, 19 kg/mol for DM, and 9 kg/mol for NM (Fig. 3b). Moreover, analysis also confirmed the absence of protein. In summary, the peak at 9.3 mL can be assigned to unfolded, detergent-free Mistic, whereas the peaks at higher elution volumes represent protein-free detergent micelles; these data are compiled in tabular form in Table 1.

**Fig. 5:**
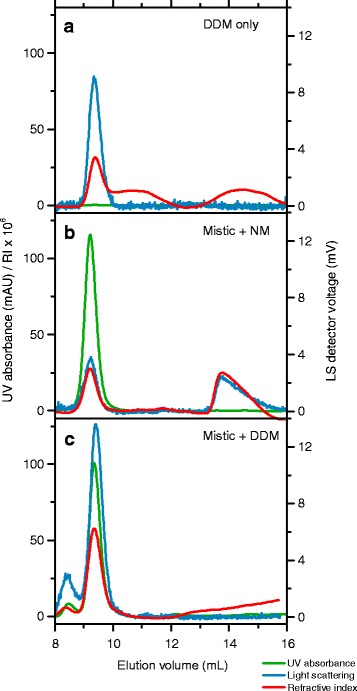
SEC as monitored by UV absorbance, refractive index, and light scattering of Mistic solubilised in DDM or NM and of protein-free DDM micelles in the presence of 6 M urea. UV absorbance and RI values as well as the voltage of the LS detector are depicted as functions of elution volume. **a** 6.08 mM DDM without protein gives rise to a single peak at ~9.3 mL in both RI and LS signals but shows no UV absorbance. **b** Mistic solubilised in 29.6 mM NM produces two peaks, namely, one at 9.3 mL representing unfolded protein and a second one at ~13 mL reflecting protein-free detergent micelles. **c** Mistic solubilised in 6.08 mM DDM reveals only a single peak at ~9.3 mL in all three detectors. 1 mg/mL Mistic, 50 mM Tris, pH 7.4, 50 mM NaCl, *c*_det,sample_ = CMC_8 M urea_ + 5 mM, *c*_det,buffer_ = CMC_buffer_ + 2 mM, flow rate 0.4 mL/min, RT

By contrast, standard analysis of data obtained on Mistic in the presence of DDM under denaturing conditions failed because it would result in physically unrealistic protein and detergent masses. Instead, the only peak in the Mistic/DDM chromatogram under denaturing conditions can be explained by detergent-free Mistic and protein-free DDM co-eluting as two independent species. This becomes obvious by comparing, on the one hand, the chromatogram of protein-free DDM micelles at 6 M (Fig. 5a) and, on the other hand, that of unfolded Mistic in the presence of the other alkyl maltoside detergents such as NM (Fig. 5b). Unfortunately, both DDM micelles and unfolded Mistic elute at ~9.3 mL, that is, at the position at which the only peak appears in the Mistic/DDM chromatogram under denaturing conditions (Fig. 5c). Although this prevents quantitative analysis of the two species following the standard protocol, a more sophisticated analysis procedure relying on a linear combination of detergent-free Mistic traces obtained from Mistic/NM measurements and protein-free DDM traces can overcome this obstacle, demonstrating that, under unfolding conditions of 6 M urea, Mistic is detergent-free in the presence of DDM [11].

On a more general note, it is important to keep in mind that complex solvent mixtures such as concentrated denaturant solutions could potentially introduce additional complications arising from binding of one solvent component to or its preferential accumulation in the vicinity of the scattering particles. In the examples discussed here, for instance, urea is expected to preferentially interact with protein and detergent moieties, which could affect both effective molar masses and refractive indices. However, our finding that triple-detection SEC yields, within experimental error, the correct molar mass of BSA in the presence of 6 M urea (data not shown) argues against a significant contribution from such effects in the present case.

## Conclusions

Triple-detection SEC is a straightforward and robust technique for analysing detergent-suspended membrane-protein samples in terms of PDC composition and oligomeric state. Caution has to be taken in special cases, for instance, in the presence of co-eluting species. Because triple-detection SEC provides averaged mass values, simultaneous passage of several co-eluting species through the detection volumes of UV, LS, and RI detectors adversely affects data analysis. For non-absorbing detergents, the protein mass can still be determined accurately, but detergent masses tend to be overestimated. If nominal or experimental data for (some of) the pure species are available, the contributions of protein and detergent to the three signals can be distinguished, so that determination of both masses is possible even under such challenging conditions.

## Methods

### Experimental design

#### General

We prepare buffers for SEC and LS experiments from ultrapure water having a resistivity of >18 MΩ cm as provided by, for example, a Millipore filtration system (Merck Millipore). All buffers are filtered through a 0.22-μm filter with the aid of a vacuum filtration system before addition of detergent. This helps avoid both clogging of the SEC column and scattering artefacts. In the course of an SEC run, the applied sample is diluted, which simplifies data analysis because no intermolecular interactions have to be considered [38, 39]. However, precautions need to be taken to avoid dilution of the detergent below its CMC, which would result in aggregation and precipitation of the protein. To ensure the presence of detergent micelles during all stages of the experiment, we typically use 2 mM and 5 mM micellar detergent in buffer and sample, respectively. Some co-solutes and co-solvents such as denaturants increase the CMC [36]; therefore, buffer and sample detergent concentrations have to be adapted under denaturing conditions. We determine the buffer’s refractive index and the refractive index increment, d*n*/d*c*, of the detergent in this buffer using a table-top refractometer. Because of the wavelength dependence of the refractive index [40], it is important that the wavelength at which the buffer refractive index and d*n*/d*c* values are obtained is close to that of the RI detector used in triple-detection SEC [39]. However, differences within the wavelength range typically employed (i.e., 630–690 nm) are negligible [30].

#### Concentration determination

For concentration determination based on the RI signal, knowledge of the refractive index increment d*n*/d*c* of all species of interest is essential. For proteins, literature values of 0.185–0.187 mL/g [28, 31] are often used. However, the d*n*/d*c* value depends on a number of factors, including buffer composition, wavelength, and temperature. The software SEDFIT (http://www.analyticalultracentrifugation.com/default.htm) contains a convenient tool providing a more accurate estimate of the d*n*/d*c* values of proteins on the basis of their amino acid composition [41]. For some detergents, literature values are available [42, 43]; for more complex buffer systems or uncommon detergents, d*n*/d*c* values can be determined as described in the procedure below. We typically use 10–15 different detergent concentrations distributed both above and below the detergent’s CMC, with the exact values depending on the detergent used. For concentration determination based on UV absorbance, the extinction coefficient has to be known. Extinction coefficients for proteins can also be estimated from amino acid compositions with the aid of SEDFIT [44] or online tools such as ProtParam (http://web.expasy.org/protparam/). For some proteins, however, the extinction coefficient estimated from primary structure differs significantly from the experimental value; in such cases, the extinction coefficient has to be determined experimentally for correct mass calculation. All detergents mentioned in this protocol do not absorb significantly in the UV range, but some detergents do, including the popular Triton series. In these cases, the extinction coefficient of the detergent has to be considered in data analysis. If no literature values are available, the extinction coefficient can be determined from UV absorbance spectra recorded at different detergent concentrations, analogously to the procedure for d*n*/d*c* determination described in the protocol part below.

#### Sample

To ensure good separation by SEC, the sample volume should be as small as possible and should not exceed the maximum volume provided by the manufacturer (typically, 0.5–2 % of the total column volume). We usually apply 50–100 μL of sample to a column of ~24 mL.

#### Calibration

When triple-detection SEC is used to determine molar masses, no system calibration for relating molar mass to elution volume is needed. Nevertheless, running a sample protein of known molar mass may be used to calibrate the system for peak broadening effects, which is caused by offsets in elution time among different detectors, volume differences among measurement cells of different detectors, and normalisation of all LS signals to the 90° signal [30]. Peak-broadening and interdetector-delay corrections have to be performed once after the system has been set up and after each change affecting detector sequence or interdetector volumes (i.e., length or diameter of tubing or flow cells). The detector with the broadest signal peak should be chosen as the reference detector for peak-broadening correction. This is usually the RI detector, which also contributes the most to additional interdetector signal broadening. Therefore, it is preferable to install the RI detector as the last detector in line. Normalisation of all LS signals to the 90° signal has to be performed for a newly installed LS detector or after exchanging the detector’s flow cell. We also recommend normalisation of the LS detector each time a new solvent is used that has a refractive index significantly different (~10 % deviation) from that of the solvent for which the last normalisation was performed.

#### Solvent refractive index

Knowledge of the refractive index of the solvent not only is important for deciding when new calibration is required but also is indispensable for data analysis. According to Equation (3), the optical constant *K* is proportional to the square of the refractive index of the solvent *n*_0_. In the above examples, with a buffer refractive index of ~1.33 in the absence of urea and ~1.38 in the presence of 6 M urea, disregarding this difference would result in systematic errors in the values of the derived molar masses of the PDC and the protein of ~8 % and, by error propagation, a larger error in the molar mass of the detergent. Solvent refractive indices can be measured using a table-top refractometer operating at a wavelength identical to or, at least, close to that of the online LS and RI detectors. Additionally, some online RI detectors can determine not only the differential RI but also the absolute RI of the solvent. The value of the solvent refractive index can be corrected before data analysis by changing the experiment configuration referring to the solvent settings in ASTRA.

#### Instruments

The protocol presented here was developed for an 1100/1200 Agilent HPLC system fitted with a G1365B UV absorbance detector and extended by a miniDAWN TREOS light scattering detector and an Optilab T-rEX refractive index detector, both from Wyatt Technology (see Equipment). Measurements were controlled through the programs ChemStation and ASTRA V from Agilent Technologies and Wyatt Technology, respectively. Note, however, that PDC composition can be determined with the aid of any setup providing triple-detection by UV absorbance, LS, and RI, and data analysis can be performed with any spreadsheet program [45] following the procedure for basic LS analysis.

### Materials

- Bovine serum albumin (BSA) (Carl Roth, cat. no. 8076, purity >98 %)
- Ethylenediamine-*N*,*N*,*N*’,*N*’-tetraacetic acid (EDTA) (Carl Roth, cat. no. 8040, purity >99 %)
- KCl (Sigma, cat. no. 9333, purity >99.0 %)
- LDAO (Sigma, cat. no. 40234; purity >99.0 %, molar mass 229.4 g/mol).
- NaCl (VWR, cat. no. 27810, purity >99.5 %)
- Tris buffer (Carl Roth, cat. no. 5429, purity >99.9 %)

### Equipment

- Buffer vacuum filtration system (Carl Roth, cat. no. XT09.1) equipped with filter paper with 0.22-μm pores (Sartorius, cat. no. 18407-50-N).
- Liquid-chromatography system; the system should include a pump providing a stable flow rate, a degasser unit, and a UV absorbance detector; available, for example, from Agilent Technologies, Bio-Rad Laboratories, Buck Scientific, GE Healthcare, Hitachi, Jasco Analytical Instruments, Perkin Elmer, Shimadzu Scientific Instruments, Thermo Scientific, or Waters Corporation.
- LS detector; in principle, a detector employing a single scattering angle is sufficient for proteins with molar masses up to ~10^6^ g/mol. For optimising the signal/noise ratio, a multi-angle LS detection system with at least three detection angles is advantageous. LS detectors are available, for example, from Malvern Instruments or Wyatt Technology.
- Refractometer, for instance, Abbemat 500 from Anton Paar or other table-top refractometer providing at least 5 digits.
- RI detector, for example, OPTILAB T-rEX from Wyatt Technology.
- Size-exclusion column, preferably with a wide separation range (e.g., 3–70 kg/mol such as Superdex 75 10/300 GL from GE Healthcare) to ensure good separation of PDCs and protein-free detergent micelles; available from Agilent Technologies, GE Healthcare, Tosoh Bioscience, and others.
- Table-top centrifuge; Eppendorf centrifuge 5340 R or other centrifuge allowing centrifugation of samples with volumes of several millilitres.
- Dialysis membrane with a molecular-weight cut-off (MWCO) of 12–14 kg/mol (e.g., Spectrum Laboratories, cat. no. 132706)
- 5-mL QuixSep Micro Dialyzer capsules (Carl Roth, cat. no. 0671.1)
- Screw cap with septum suitable for screw-top vial (Carl Roth, cat. no. LC13.1)
- 1.5-mL screw-top vial (Carl Roth; cat. no LC03.1)
- ASTRA software, version V (Wyatt Technology) or other software for operating LS and RI detector and recording data.
- ChemStation software package (Agilent Technologies) or other software for operating HPLC system and UV detector.
- Spreadsheet program such as Microsoft Excel, Libre Office, Open Office, etc. if required for more sophisticated analysis (see Results and Discussion).

### Reagent setup

The reagent setup described below is applicable to system calibration with BSA and characterisation of OmpLA/LDAO complexes as detailed in “Separation and characterisation of PDCs and protein-free micelles” and shown in Fig. 3. Note that system calibration can be performed with any sample providing a well-resolved peak. For other protein/detergent combinations, the reagent setup will have to be adjusted to meet specific requirements. Use of buffer stocks is recommended for preparation of buffered solutions containing different detergent concentrations.

**Solution A:** Prepare 1.5 L buffer solution containing 50 mM Tris, 100 mM KCl, 2 mM EDTA, adjust the pH to 8.3 at RT, and filter the buffer through a 0.22-μm filter using a vacuum filtration system.

**Solution B:** Prepare 500 mL buffer containing 50 mM Tris and 50 mM NaCl and adjust the pH to 7.4 at RT. Filter the buffer through a 0.22-μm filter using a vacuum filtration system.

From these buffer stock solutions, all required buffers are prepared by addition of concentrated detergent or protein and detergent stocks.

For d*n*/d*c* determination, a detergent concentration series is needed. To this end, a detergent (here, LDAO) stock solution is used to minimise non-systematic errors.

**Solution C (LDAO stock solution):** Weigh 57.35 mg LDAO and dissolve it in 10 mL solution A to a final concentration of 25 mM LDAO.

System calibration is performed using the BSA monomer peak in the absence of detergent.

**BSA solution:** Weigh 2 mg protein and dissolve it in 2 mL solution B. To remove aggregates and dust, filter the protein solution through a 0.22-μm syringe filter or centrifuge it for 10 min at 20’000 *g*.

Because refolded OmpLA is usually stored in a buffer different from the one used in the protocol below, buffer composition as well as protein and detergent concentrations have to be adjusted prior to triple-detection SEC measurements.

**OmpLA stock solution:** Refolded OmpLA (typically at a concentration of 3–4 mg/mL) is dialysed to adjust the detergent concentration to 5 mM and to complex traces of Ca^2+^ that would induce dimerisation of OmpLA. To this end, proceed as follows:

**Solution D (dialysis buffer):** Weigh 573.5 mg LDAO and dissolve it in 500 mL solution A to a final concentration of 5 mM LDAO.

**Solution E (running buffer): **Weigh 458.8 mg LDAO and dissolve it in 500 mL solution A to a final concentration of 4 mM LDAO.

**OmpLA sample: **Dialyse at least 1 mL of the OmpLA stock solution with the aid of a Micro Dialyzer capsule fitted with a dialysis membrane with a cut-off of 12–14 kg/mol against a 100-fold excess volume of solution D overnight at RT. Determine the final protein concentration and prepare a 500-μL sample of ~3 mg/mL OmpLA by dilution with solution D. To remove aggregates and dust, centrifuge the sample for 10 min at 20’000 *g* and 10 °C.

### Protocol

#### Determination of refractive index increment

1| From solutions A and C, prepare an LDAO dilution series at 15 different concentrations with 1 mL of each concentration (e.g., 25, 20, 15, 10, 9, 8, 7, 6, 5, 4, 3, 2, 1, 0.5, and 0 mM) (30 min).

2| Record the refractive index of each sample and determine d*n*/d*c* using either one of the following two procedures (2-3 h).

(A) **Use of a standalone table-top refractometer.** Use a table-top refractometer measuring at a wavelength close to that of the online LS and RI detectors.
  (i) Set the measurement temperature to 20 °C.
  (ii) Wipe the measuring prism with ultrapure water to remove dust and contaminations.
  (iii) Apply ~500 μL sample, starting with pure solvent, and wait until the signal has stabilised. It is of critical importance to wait until the displayed value has stabilised; for denaturant-containing solutions, in particular, this may take some time (i.e., 10–15 min).
  (iv) Record the displayed RI value and continue with the next concentration.
  (v) Correct the refractive index at each LDAO concentration by subtracting the refractive index of solution A (*n*_0_) to obtain Δ*n = n*–*n*_0_*.*
  (vi) Plot Δ*n* versus the detergent concentration in g/mL.
  (vii) Perform a linear regression of the data using a spreadsheet program such as MS Excel [45]. The slope provides the d*n*/d*c* value in mL/g.
(B) **Use of the OPTILAB T-rEX in batch mode.** At this point, it is necessary that the RI detector is connected to the HPLC pump by a loop of 0.5–1 mL, bypassing any column or other detector, or to a syringe pump providing a flow rate of 0.1–0.2 mL/min.
  (i) Turn *Purge* on and flush the reference and measurement chambers with ultrapure water for ~10 min.
  (ii) Turn *Purge* off and monitor the baseline. When the baseline signal is stable, zero the RI detector by selecting *Zero* in the detector *Main* screen.
  (iii) Prepare for data acquisition with the ASTRA software by opening a new experiment using the template *Batch (determine dn/dc)*.
  (iv) Start data acquisition by running the experiment and introduce pure solvent as first sample. Wait until the baseline signal is stable. Manual sample application is preferred over use of an autosampler because the former is more flexible in terms of volume applied and time allowed for signal stabilisation.
  (v) During data collection in ASTRA, introduce the series of LDAO concentrations starting with the lowest concentration. For each concentration, wait until the signal stabilises and reaches a plateau before you apply the next sample.
  (vi) After the sample with the highest LDAO concentration has been measured, inject solution A (i.e., pure solvent) again to re-establish the baseline. When the baseline is stable, stop data acquisition.
  (vii) In the *Baselines* section, define a baseline by choosing the pure solvent blank. Left-click on the left-hand side of the plot and drag a line from the blank plateau at the beginning to the blank plateau at the end of the acquisition.
  (viii) Define a peak for each plateau region, but do not select a peak for the blank injection. In the table at the *Refractive Index* node, enter the concentration for each sample in the row entitled *Concentration (g/mL)*.
  (ix) Apply your settings by clicking *OK*; now, the d*n*/d*c* value is provided in the *Report* section.

Determination of d*n*/d*c* values as described above can be performed independently of all other steps.

#### System calibration (bench time 2 h, additional 4 h of automated run)

3| **Equilibration of the system.** Equilibrate the Superdex 75 10/300 column (dimensions 1.0 cm × 30 cm, total volume ~24 mL) and the detectors by running solution B at a flow rate of 0.5 mL/min for ~180 min. Make sure that the RI detector is in purge mode to exchange the buffer in the reference and measurement chambers.

4| Turn the UV lamp and the LS laser on and wait for at least 20 min before starting data acquisition.

5| To ensure equilibration, verify that all three detectors (i.e., UV, LS, and RI) show stable baseline signals towards the end of the equilibration period.

6| Disable *Purge* at the RI detector.

7| **Configuration of ASTRA experimental settings.** Use either one of the following two procedures.

(A) Create a new experiment in the ASTRA software using a template including data collection of all three detectors (i.e., UV, LS, and RI) as well as the procedures *Interdetector Delay*, *Band Broadening*, and *Normalization*.
(B) Create a new experiment template.
  (i) Open a new experiment using the system template *online*.
  (ii) Add a new detector by choosing *Configuration* → *Edit Configuration* in the *Instruments* section. In the *Add* line, choose *Generic UV instrument*.
  (iii) Set the *UV Response Factor* to 1 (in general, you will find the right value for your detector in the detector manual) and disable *Band Broadening*.
  (iv) Add two *Fluid connections* and one *Aux channel connection*, representing real fluid connections and a data connection from the UV detector, respectively. Proceed as when adding a new detector, but choose the *Add* → *Browse* command in the *Connection* section. For the first fluid connection, specify the *Injector* and the *Generic UV instrument* as *Source* and *Destination Device*, respectively. The second fluid connection must contain the *Generic UV instrument* and the *miniDAWN TREOS* as *Source* and *Destination Device*, respectively. For the Aux connection, the *Generic UV instrument* must be specified as *Source Device* and the *LS detector* as *Destination Device*. Additionally, specify the respective data port number for the Aux channel; in case of doubt, check your computer’s settings for the correct port number.
  (v) Change the LS device to *miniDAWN TREOS* if another LS device is specified.
  (vi) Save the configuration as *SEC_SLS_Calibration* by choosing *Save As Template*.

8| Under *Basic collection*, set *Duration* to 60 min and *Collection Interval* to 0.125 s. Make sure to enable *Trigger on Auto-Inject*.

9| Save the experiment.

10| **Create an acquisition method in ChemStation.** From the *Method* menu, select *Edit Entire Method*. Enable *Method Information* and *Instrument*.

11| In the *Method Information* window, you can enter information about the intended use of the method (e.g., method for system calibration), buffer composition (50 mM Tris, 50 mM NaCl, pH 7.4), SEC column specification (Superdex 75 10/300), and flow rate (0.5 mL/min). This has no influence on the experimental settings but helps in identifying the method later on.

**Fig. 6:**
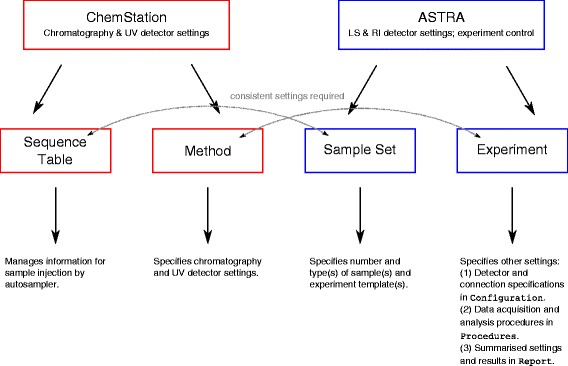
Scheme of control software. The main features of ChemStation and ASTRA and the menus for controlling detectors, data acquisition, and data analysis are depicted together with the interfaces between the two programs

12| Specify method settings in the respective dialogue as given in Table 2. The *Stop Time* must be the same as the *Duration* in ASTRA’s experimental settings; otherwise, data collection for the second and following injections cannot be triggered by the injection signal. See Fig. 6 for an overview of the interplay between the ChemStation and ASTRA software packages. Accept changes by clicking *OK*.

**Table 2:** Method settings in ChemStation for system calibration

Pump Parameter	
Flow	0.5 mL/min
Stop Time	60 min
Solvent A	100 %
Pressure Limits Max	system pressure + 18 MPa
Injector	
Standard Injection	Enable
Injection Volume	100 μL
DAD Parameter (UV diode array)	
Signals Store	Enable 280 nm
Stop Time	As pump
Peakwidth	1 s
Slit	4 nm

13| Save your method as a new file with the name *SEC_SLS_Calibration*.

14| From the *Sequence* menu, select *Sequence Table*.

15| Fill the table with the information given in Table 3. The sample volume specified in the sequence table must be the same as the injection volume in the method used. Otherwise, the sequence table setting overwrites the method setting.

**Table 3:** Sequence table settings in ChemStation for system calibration

Location	Position of your sample in the sampling tray
Method	SEC_SLS_Calibration
Injection (number of replicates of the sample that must be run)	1
Sample volume	100 μL

16| In the sequence menu, select *Sequence Parameter*.

17| Enter your name as operator and specify the prefix as *SEC_SLS_Calibration*, extended by the counter serving as file name, and the subdirectory in which your data should be stored. If you omit this step, you will overwrite older files.

18| Save your sequence as *SEC_SLS*.

19| Transfer your BSA sample into a screw-top vial with septum, place it into the sample tray, and make sure you put the tray back in place correctly.

20| **Start the experiment in ASTRA.** In the *Experiment* menu, select *Run*. *Waiting for auto-injection signal* will be displayed in the *Collection plot*. It is crucial to always start the sequence in ASTRA before starting the method in ChemStation; otherwise, data collection cannot be triggered by the auto-injection signal.

21| **Start the run in ChemStation.** Click the *Start* button to start the method. Select *Yes* when asked whether you want to save changes you made to the method.

22| After the run is completed, open your datasets in ASTRA. Choose the *SEC_SLS_Calibration* dataset and re-run it by selecting *Run* in the *Manage* option of the selected file.

23| **Baseline correction.** Go to *Baselines* and enable the LS 2 trace. Set your baseline by left-clicking in the blank region at the beginning of the trace before (i.e., to the left of) the peak(s) and dragging to the blank region at the end of the trace (i.e., to the right of the peak(s)). Click *Auto Baseline* to transfer your selection to all other traces.

24| Visually inspect the baseline settings in all traces and change them if the displayed baseline cuts peaks or proceeds below the background level of the signal. Click *Apply* to re-run the analysis sequence with new baseline settings.

25| **Interdetector delay correction.** Go to *Interdetector Delay* and select the main region of the BSA monomer peak at an elution time of ~19 min. Click *Determine Delays*. The procedure provides the signal delays of the LS and RI detectors referenced to the UV absorbance detector in millilitres.

26| Apply the value obtained for the UV–LS connection in the *Volume* row of the *Fluid connection* configuration for these two detectors. Click *Apply*.

27| To obtain the delay volume between LS and RI detectors, determine the difference between the UV–LS and the UV–RI interdetector delays and insert this difference in the *Volume* row of the *Fluid connection* between LS and RI detectors. Click *Apply*.

28| **Band-broadening correction.** Go to the *Band Broadening* procedure and select the BSA monomer peak; be careful not to choose a region affected by other eluting species. Typically, one starts halfway up the leading edge until the point where all detector signals have returned to the baseline. Choose the RI detector as *Reference Instrument* and perform the fit by clicking *Perform Fit*.

29| Examine the fit. Without despiking or smoothing, the *Instrumental Term*, which represents the additional volume introduced by the measurement cell, should not exceed 1 μL. If despiking or smoothing has been performed before, this value might be >1 μL but should still be smaller than the *Mixing Term*, which accounts for the influence of the capillary. If the *Instrumental Term* is significantly larger and matching between the peaks is not good, repeat the fit by using *Reset* and *Apply* seed values for *Instrumental* and *Mixing Term*.

30| If matching between the peaks is good, click *OK* to re-run the experiment with the new values.

31| Additionally, insert the determined *Instrumental* and *Mixing Term* in microlitres in the respective sections in the configuration sheets of the LS and UV detectors.

32| **Peak selection.** Define the peak to be analysed around the maximum of the BSA monomer peak by left-clicking into the chromatogram and dragging the borders around the region of interest. Make sure that your selection does not contain any contamination from other eluting species, particularly, the dimer fraction of BSA.

33| **Normalisation.** Select the *Normalization* procedure. Set *Peak Name* to *Peak 1* and *Radius* to 3 nm. *Normalization Type* has to be standard and *Radius Type rms*. Choose *Action Normalize*.

34| Click *Apply* to use the new normalisation values.

35| Insert the normalisation values in the *Normalization Coefficients* section of the miniDAWN TREOS configuration in the row entitled *New*.

36| Save the experiment with all changes as template under *SEC_SLS_online*. Measurements used to determine PDC composition can be performed independently.

#### Measurement of OmpLA/LDAO complexes (bench time 45 min, additional 8 h of unattended equilibration and data acquisition)

37| Determine the refractive index of solution E as described in step 2|.

38| **Preparation of the system.** Equilibrate the SEC column and the detectors as described in Steps 3|–6| but with solution E, a flow rate of 0.4 mL/min, and for 210–240 min.

39| **Configuration of ASTRA experimental template.** Open a new experiment in ASTRA based on the template *SEC_SLS_online* (see step 36|).

40| Adjust *Flow Rate* to 0.4 mL/min, *Duration* to 80 min, and *Collection Interval* to 0.5 s. Make sure *Trigger on Auto-Injection* is enabled.

41| Additionally, the changes given in Table 4 regarding pump, sample, and buffer configuration must be applied.

**Table 4:** ASTRA settings for PDC measurements

Generic pump	
Flow Rate (mL/min)	0.4
Solvent	
Reference Refractive Index	1.335 (i.e., the refractive index determined in step 37|)
Injector	
Injected Volume (mL)	0.05
Sample	
d*n*/d*c* (mg/mL)	0.1946
UV Extinction Coefficient (mL/(g cm))	2668

42| Save the changed experiment template as *SEC_SLS_PDC*.

43| **Generation of a sample set in ASTRA.** For measurements comprising more than one injection or sample, it is convenient to create a sample set instead of a single experiment in ASTRA. For this purpose, choose *Blank* in the *New* section of the *File* menu.

44| Specify the experiment template and provide sample information. To this end, follow either one of the following two procedures.

(A) Work with a general experiment template for all samples.
  (i) Select the *SEC_SLS_PDC* template from step 42| as *Default Experiment Template*. This will be applied to all samples specified in the sample set. Sample information is applied automatically.
  (ii) Specify the position of your sample in the sampling tray in the field entitled *Well*. Insert *OMPLA_LDAO* as *Name* under which data shall be saved and set the number of injections in the *Inj* field to 3. Check if the other settings are identical to the ones given in steps 40| and 41|.
(B) Work with separate experiment templates for each independent sample.
  (i) Leave *Default Experiment Template* blank and choose the *SEC_SLS_PDC* template from step 42| for each sample in the *Samples* node individually. Thus, you can specify a different experiment template for each sample.
  (ii) Specify the position of your sample in the sampling tray in the field entitled *Well*. Insert *OMPLA_LDAO* as the name under which data shall be saved and set the number of injections in the *Inj* field to 3. Set the values of the other fields to the values given in steps 40| and 41| and select *SEC_SLS_OMPLA* as *Template*.

45| Save the sample set as *OMPLA_LDAO*.

46| **Configuration of ChemStation settings.** In the method *SEC_SLS_Calibration* (see step 13|), change *Flow Rate*, *Stop Time*, and *Injection Volume* to 0.4 mL/min, 80 min, and 50 μL, respectively.

47| Save the method under the name *SEC_SLS_PDC*.

48| Continue as described in steps 14| to 18| but make sure that the number of injections in the sequence table now equals 3.

49| Transfer the OmpLA sample into a screw-top vial with septum, place it into the sample tray, and make sure to put back the tray correctly.

50| Start data acquisition as described in steps 20| and 21| but by choosing *Run* in the *Sample Set* instead of the *Experiment* menu.

Data analysis can be performed independently of data acquisition.

#### Data processing (10-30 min per experiment)

51| In the *File* menu, choose *Open* and select the experiment file *OMPLA_LDAO(001)[OMPLA_LDAO]* from step 50| to load it into ASTRA.

52| From the *Manage* menu of the experiment, choose *Add To Experiment* and select the *Analysis* procedure *Protein conjugate analysis*. Move it by drag-and-drop into the analysis sequence so it is placed before the *Fit Mass or Radius* procedure.

53| Add an additional report for the protein conjugate. To do so, go to *Add To Experiment* and open the *Result* folder. Choose *Report* and click *OK*. A new report entry is created.

54| Double-click the newly added *Report (untitled1)* item. Expand the *Template* node and select the system report template protein_conjugate_detailed from the *Report* folder in the ASTRA installation folder. In the field entitled *Description*, name the report as *Report PDC Characterisation*.

55| Set the baseline as described in steps 23| and 24|. The selected baseline range should span almost the entire elution time window to correct for fluctuations.

56| Select peaks around the maximum of the OmpLA/LDAO and the LDAO peaks at ~9 mL and ~11 mL, respectively. The detailed procedure is described in step 32|. Make sure your selection does not include parts of other peaks.

57| The table below the chromatogram displays both selected peaks. In the fields at the *LS Analysis* node, the settings given in Table 5 should be displayed. If one or more values differ from the above ones, change them accordingly.

**Table 5:** Settings for LS analysis in ASTRA

Model	Zimm
Fit Degree	1
d*n*/d*c* (mL/g)*	0.1946
UV Extinction (mL/(g cm))	2668
Modifier d*n*/d*c* (mL/g)	0.1592
Modifier UV Extinction (mL/(g cm))	0

(*) d*n*/d*c* value of the protein; here determined with the SEDFIT software tool [41]

58| Additionally, insert the detergent’s d*n*/d*c* value determined in steps1| and 2| in the field entitled *Modifier* d*n*/d*c (mL/g)* at the *Extended Parameters* node of both peaks. Click *Apply* to re-run the analysis sequence with the changed settings.

59| In the *Protein Conjugate Analysis* section, inspect the Zimm plot and the values determined for the mass of the PDC and the masses of the protein and the detergent for each data point of the selected peak range.

60| Go to the *Report* section and select *Report PDCCharacterisation*. The latter summarises all information regarding the configuration of your system, including detector settings, peak settings used for analysis, and analysis results both as weight- and number-averaged values of the molar mass of the PDC, the protein, and the detergent and as *z*-averaged values of the radius of the complexes in both peaks. As additional information, the protein fraction moment and the polydispersity are provided.

61| Check, in particular, the analysis range, the settings for the solvent refractive index d*n*/d*c*, and the absorbance values for correctness. If necessary, change settings in the appending section and apply the changes to the experiment.

62| Save the analysed experiment.

(A) Choose *Save*. The analysed experiment is saved.
(B) Choose *Save As Template*. Changes in the analysis procedure and sample settings are saved as template and can be applied to the other two data files of the measurement. Name the template *SEC_SLS_OMPLA*. Changes described in steps 52| to 54| and 58| are superseded.

All data files can be analysed independently. The experiment can be saved after any step, and analysis can be continued later.

63| Analyse the other two datasets as described above.

(A) If alternative 62|(A) was chosen above, open the datasets *OMPLA_LDAO(002)[OMPLA_LDAO]* and *OMPLA_LDAO(003)[OMPLA_LDAO]* and repeat steps 51| to 62|.
(B) If you chose alternative 62|(B), open the datasets *OMPLA_LDAO(002)[OMPLA_LDAO]* and *OMPLA_LDAO(003)[OMPLA_LDAO]*, go to *Apply Template* in the *Experiment* menu, and select the template *SEC_SLS_OMPLA* from step 62|(B). Baseline correction and peak selection have to be performed as described in steps 23| and 55|, respectively. Repeat steps 59| to 62| as described above.

### Troubleshooting advice

The most common problems encountered in triple-detection SEC and data analysis, the steps where they most likely appear, and possible solutions are summarised in Table 6.

**Table 6:** Troubleshooting advice

Step	Problem	Possible Reason	Solution
2	The refractive index increases even after long equilibration time.	High denaturant concentrations can cause detergent precipitation.	Dilute sample to lower denaturant concentration and perform experiment at this concentration.
		Denaturant precipitates.	
2B	No plateau is reached.	Loop volume is too small; hence, the applied volume does not completely fill the measurement chamber.	Install larger sample loop.
2B	Change between plateaus is not pronounced enough for reliable discrimination.	Flow rate of syringe pump is too high.	Reduce flow rate to (0.1–0.2) mL/min.
2B	Baselines are unstable.	Disconnection of syringe causes pressure changes and injection of air bubbles.	Wait for a few minutes until the baseline is stable again.
5, 21, 38, 50	RI baseline is unstable.	Too much gas dissolved in solvent.	Make sure your buffer is degassed before using it in triple-detection SEC.
		The flow rate has changed.	Use the same flow rate during system equilibration and measurement to allow the baseline to stabilise.
19–21, 50	Measurement cannot be started.	Sample tray is not placed correctly. Connection between software and LS or RI detector is lost.	Remove tray and make sure it is put back in place correctly. Close ASTRA, restart the detectors, and subsequently restart ASTRA.
21, 50	No data acquisition in ASTRA.	Method in ChemStation was started before the sample set in ASTRA was started.	Stop method in ChemStation, check that enough sample is left, and start ASTRA data acquisition before restarting the method run in ChemStation.
28	Reference detector is not the one with the broadest signal.	Wrong reference detector chosen. Inappropriate peak selection.	Make sure to choose the detector with the broadest signal. This is normally the last detector in line and should be the RI detector. Make sure you set the peak boundaries from halfway up the peak to the point where all detector signals have returned to baseline.
55	Baseline cuts peaks to be analysed.	High denaturant or salt concentrations cause baseline instabilities.	Set baselines manually for each detector individually, such that the flanks of peaks of interest essentially reach baseline level without being cut or shifted upwards.
59	Systematic deviation from linearity of one of the LS detectors.	Detectors are not normalised correctly.	Check detector normalisation values. If necessary, repeat normalisation. If the sample analysed reveals a unimodal particle size distribution, normalisation can be done using the actual measurement according to the procedure described in step 33|.
59	Data points cannot be fitted with a linear fit.	Scattering particles are large (i.e., >50 nm), and, thus, the Zimm plot is significantly curved.	Select a different fit model in the peak section.
59	Molar mass plot is bent upwards or downwards within the analysed region (smiley or anti-smiley effect).	Band-broadening correction is incorrect.	Check settings for instrument and mixing terms; if necessary, repeat band-broadening correction for current solvent system.
60	The results obtained are far from expected or reasonable values.	Incomplete separation of different species.	Install a different SEC column that is able to separate the species of interest. Try manual, more complex analysis algorithms that are able to distinguish contributions from different species (see Results and Discussion).
60, 63	Analysis is not possible. Molar masses are displayed as N/A in the final report.	Baseline correction, peak selection, or constants needed for analysis were not adapted to the system being analysed.	Check if baseline settings and peak selection are correct and if the saved constants correspond to the system you are analysing.

## Abbreviations

AUC: analytical ultracentrifugation
BSA: bovine serum albumin
CMC: critical micellar concentration
DDM: *n*-dodecyl-β-D-maltoside
DLS: dynamic light scattering
DM: *n*-decyl-β-D-maltoside
EDTA: ethylenediamine-*N*,*N*,*N*’,*N*’-tetraacetic acid
LDAO: lauryldimethylamine *N*-oxide
LS: static light scattering
MWCO: molecular-weight cut-off
NM: *n*-nonyl-β-D-maltoside
OmpLA: outer membrane phospholipase A
PDC: protein/detergent complex
RI: refractive index
RT: room temperature
SDS-PAGE: sodium dodecyl sulphate polyacrylamide gel electrophoresis
SEC: size-exclusion chromatography
UM: *n*-undecyl-β-D-maltoside
UV: ultraviolet
